# Associations between Subjective and Objective Measures of the Community Food Environment and Executive Function in Early Childhood

**DOI:** 10.3390/nu12071944

**Published:** 2020-06-30

**Authors:** Lindsey M. Bryant, Heather A. Eicher-Miller, Irem Korucu, Sara A. Schmitt

**Affiliations:** 1 Human Development & Family Studies, Purdue University, 1202 W. State Street, West Lafayette, IN 47907, USA; saraschmitt@purdue.edu; 2 Department of Nutrition Science, Purdue University, 700 W. State Street, West Lafayette, IN 47907, USA; heicherm@purdue.edu; 3 Yale Center for Emotional Intelligence, Yale University, 350 George Street, New Haven, CT 065011.3, USA; irem.korucu@yale.edu

**Keywords:** food access, executive function, preschool children, community food environment

## Abstract

The present study utilized a cross-sectional design to assess whether two indicators of the community food environment, parent perceptions of the community food environment (i.e., as assessed by parent reports of access to, availability, and affordability of foods) and limited food access (via census data), were related to executive function in preschool children. Children were recruited during the 2014–2015 academic year from Head Start and community-based preschools (*N* = 102) and children’s executive function ability was tested using the Head–Toes–Knees–Shoulders task. Multiple linear regression analysis was used, as well as adjusted standard errors to account for clustering at the classroom level. Parent reports of their food environment were significantly related to children’s executive function, such that children living in higher quality community food environments had better executive function. In contrast, limited food access using census data was not significantly related to executive function. The results suggest that parent reports of the community food environment in early childhood may contribute to young children’s cognitive outcomes more so than being in a limited food access area, as these data may not represent individual behaviors or capture the variability of the accessibility and affordability of healthy foods. Policy makers should consider correlations between the food environment and early executive functioning when developing new community health/wellness legislation.

## 1. Introduction

The consumption of foods with a low nutrient density is an important correlate of well-being throughout life and a modifiable risk factor for chronic disease and obesity, which may originate in childhood [1,2,3]. Previous studies suggest that not eating a healthful diet, adhering to the Dietary Guidelines for Americans (DGA) recommendations, may lead to adverse health outcomes, including iron-deficiency anemia, acute infection, chronic illness, and developmental and mental health problems among children [3,4,5,6]. Thus, determining the barriers to health and nutrition among United States (U.S.) children is critical to improving child nutrition and health in both the short and long term [7,8]. The community food environment, conceptualized as the reported availability, affordability, and accessibility (e.g., available transportation) of grocery stores or other entities that sell foods that promote a healthful diet via the DGA [3], has received consideration as a possible determinant of dietary intake and may potentially be associated with health and nutritional outcomes [9,10]. Evidence that U.S. children and adults with access to foods that most children do not consume enough of, such as fruits and vegetables, fare better in terms of their physical health and development compared to those without such access, supports recognition of the community food environment as a potential barrier to health and nutrition [3,11,12].

However, most of the research examining the community food environment in the context of child health and nutrition focuses on the consumption of fruits and vegetables and dietary intake [13,14,15,16], but does not consider a potential link between the community food environment and cognitive outcomes. Alternatively, the research related to cognitive outcomes has explicitly evaluated the role of nutrition and obesity (e.g., body mass index (BMI)) in older children and adults on cognition and has not explored the association of the community food environment with cognitive development in young children [16,17,18,19]. Thus, very little is known about the potential association between the community food environment and cognition among children. Further, no studies have examined the relation between the community food environment (as assessed both subjectively via parent reports and objectively via census data) and executive function in early childhood, thus warranting further exploration.

Executive function (EF) is defined as the ability to flexibly control automatic thoughts and responses in order to remain goal oriented [20,21] and is considered to have three integrated, cognitive components [22,23]: cognitive flexibility, inhibitory control, and working memory. EF in early childhood is considered an important predictor of short- and long-term health, social–emotional, and academic outcomes [21,24,25,26,27,28]. For instance, children with stronger EF during the preschool years (3−5 years of age) have lower BMIs [25] and demonstrate better social–emotional competence, school readiness, and subsequent academic achievement (e.g., literacy and math [21,26,27,28]). Furthermore, the preschool years are considered a sensitive period for the development of EF due to structural changes in the prefrontal cortex [20,29]. Thus, EF in this developmental stage may be more susceptible to environmental influences. This may be particularly true for nutritional deficiencies during the preschool period [30,31]. Nutrients provide the necessary components for developmental processes in the brain that impact cognitive development (e.g., neuronal/glial metabolism, myelination, enzyme systems), and deficits to these processes may have a larger impact when the brain is rapidly changing [30,31]. Thus, the community food environment, particularly the availability and affordability of healthful foods, may support EF development in preschool; however, to date, no studies have explored this association.

### An Ecological Systems Perspective

The ecological systems perspective illustrates the barriers and opportunities of the community food environment [7,8,32,33]. This perspective posits that, although development is impacted by multiple levels of children’s environments, proximal contexts or microsystems (including barriers to healthy food in the home and neighborhood contexts) are the most critical, and often interact to influence developmental outcomes, such as EF [32]. Afshin and colleagues use this perspective to explain an individual’s relationship with health and nutrition using a series of microsystems ranging from the most distal, like global impact (e.g., global food availability, international food standards) to sociocultural influences (e.g., social support, social class, social culture norms, social cohesion) to characteristics of an individual (e.g., age, sex, nutritional knowledge, and skills [7]). This framework acknowledges the multiple factors that impact health and nutrition while specifically focusing on associations between individuals and their environments. Barriers to health and healthy diets within the larger ecological system can lead to nutrition risk, which may impact parts of the developing brain that are associated with EF skills [30], thus impacting early EF ability. Though these barriers likely apply more directly to adults, children’s food consumption is influenced by parenting behaviors, including parental fruit and vegetable, fat, and soft drink intake [34,35], as well as parental feeding style and eating practices [8]. If parents experience barriers related to the availability and affordability of foods, their children may consequently be impacted when these barriers are linked with developmental trajectories [11,12]. Furthermore, parent experiences and perceptions of the community food environment (via parent reports of access to, availability, and affordability of foods) may be particularly important for evaluating access to healthful foods, relative to more distal assessments of the community food environment (i.e., census tract data of limited access). This may be true given that parents are embedded in their communities, likely understanding the nuances of their access and affordability. In support of this hypothesis, previous literature has identified that adults are able to accurately assess their access to healthful foods, whereas food desert status (as indicated by U.S. Department of Agriculture (USDA) and geographic information systems (GIS) data at the census tract level) does not predict where (e.g., nearest store) and how (e.g., shopping frequency) individuals obtain healthful foods [36]. These discrepancies may be a result of certain assumptions made when evaluating access using census tract data (e.g., assuming individuals buy from the closest stores [37]). Therefore, knowledge of how the community food environment (perceptions or limited access) is related to child development may inform public health programs and policies.

Therefore, the primary aim of this study is to assess whether two indicators of the community food environment, parent reports of the food environment (e.g., access to, availability, and affordability of foods) and limited food access (via census data), are related to EF in children who are three to five years old. We included a subjective (parent reports) and objective measure (census data on limited food access) of the community food environment to test whether differential associations would emerge between these indicators and EF skills. We expected that children with better community food environments across both indicators would have higher scores on an EF task compared with those with worse community food environments.

## 2. Materials and Methods

### 2.1. Study Design and Participants

Participants of this cross-sectional study included 102 children (52% female) and one of their parents recruited from 25 Head Start (a federal U.S. preschool program for children from families with low incomes (according to federal U.S. Poverty Guidelines) that provides early child care and education) or center- and community-based preschools located in the central and western regions of a Midwestern state in the U.S. Children ranged from 40 to 66 months (Mean [M] = 53.57, SD = 5.42), and 51% of children were enrolled in Head Start classrooms. Parents’ highest level of education ranged from 8th grade to doctoral degree, and approximately 50% of the sample had a high school degree or less. Refer to Table 1 for full descriptive and demographic information.

### 2.2. Procedures

Recruitment occurred during the 2014–2015 academic year after the study was approved by a university Institutional Review Board. Preschools were selected using convenience sampling. Parents of all children within the target age range of 3–5 years old at participating preschools were sent a letter describing the study, inviting them and their child to participate. Written consent was obtained from parents/primary caregivers prior to participation. Children in the study did not have any known pervasive developmental disorders or have severe auditory or visual impairments that were not corrected, and all children were English language speakers who were able to participate in assessments that required an age-appropriate level of English proficiency. All data were collected in the preschool year at one time point. Parents self-reported demographic and family characteristics (e.g., home learning environment) on paper surveys, and children were interviewed in a quiet space in their classrooms for the direct assessment of EF. All participants received a $20 gift card and children received stickers after completing assessments.

### 2.3. Measures

#### 2.3.1. Food Environment

Parent reports of the food environment were assessed using a four-item survey that included the following items: “My family has access to a grocery store”; “There is public transportation to the grocery store”; “Is healthy food available in your community?”; “Is healthy food affordable in your community?” Responses were scored 0 for “no” and 1 for “yes” and scores were summed (range 0–4) to create the independent variable for use in analysis. Lower scores indicated a poorer food environment based on the U.S. Department of Agriculture’s (USDA) definition of food access [38]. Researchers created this parent-report tool because a brief measure, quantifying perceptions of access, transportation, and healthy food availability and affordability was not available [8,11]. The simplicity of the measures (four items), which is similar to other measures that have been used in previous studies [36,39,40,41,42], affects the moderate internal reliability for this novel measure (α = 0.50).

#### 2.3.2. Limited Food Access

Census tract information for each residential address (parent-reported) in the sample was obtained from the American Community Survey (ACS) data from the U.S. Census Bureau [43], which uses geographical information systems (GIS) software to map addresses to a specific census tract that corresponds with an address. We used tracts from the 2015 data set, the year the data were collected. After identifying what tract families were in, we used a low-access tract variable from the USDA Food Access Research Atlas data set [44]. The Food Access Research Atlas data flagged a tract as low access if at least 500 people within the tract, or 33% of the population, were living more than ½ mile (urban areas) or 10 miles (rural areas) from the nearest supermarket, supercenter, or large grocery store. In the USDA’s 2017 report, a directory of supermarkets, supercenters, and large grocery stores within every state was derived by merging the 2015 Store Tracking and Redemption System (STARS) directory of stores authorized to accept Supplemental Nutrition Assistance Program (SNAP) benefits and the 2015 Trade Dimensions TDLinx directory of stores. The block-level population data were derived from the 2010 Census of Population and Housing. A score of 1 indicated a tract was low access and a score of 0 indicated the tract was not considered low access.

#### 2.3.3. Executive Function

Children’s executive function was assessed using the Head–Toes–Knees–Shoulders task (HTKS) [45]. The HTKS is a behavioral measure that directly taps into all three components of EF (cognitive flexibility, inhibitory control, working memory), and is typically used with children aged 3–7 [45]. In the practice round, children are first asked to respond by following the directions normally (e.g., “Touch your head”), and then they are asked to respond in the opposite way (e.g., children are asked to touch their heads when the research assistant says, “Touch your toes”). The testing portion consists of 30 items (three sections of ten), and the sections get increasingly complex as the child progresses. In order to progress to the second section, a child has to receive a score of at least 4 on the first section, and similarly, in order to progress to the third section, a child has to receive a score of at least 4 on the second section. Each correct response is worth two points, making the range of possible scores 0–60. Each item is scored as 0 (incorrect), 1 (self-correct), or 2 (correct). The total score of the test is the sum of all the correct items. This task takes approximately 5–10 min to complete. The interrater reliability, scoring agreement, and test–retest reliability is high and shows strong predictive validity [45]. The HTKS has moderate to strong effect sizes, predicting achievement levels and gains across multiple studies in pre-k and kindergarten-aged children [45,46,47].

#### 2.3.4. Covariates

The characteristics of children and parents, including child sex (1 = female), child age (range: 40–66 months), race/ethnicity (0 = White/Caucasian, 1 = non-White/Caucasian) and parent education (1 = 8th grade or less, 2 = some high school, 3 = GED, 4 = high school diploma, 5 = some college, 6 = associate’s degree, 7 = bachelor’s degree, 8 = master’s degree, 9 = doctoral/postgraduate degree) were also assessed in the participant survey. See Table 1 for details on response categories. The home learning environment was included as a covariate in order to capture other potential confounders that may be related to EF development [48]. Thus, the home learning environment was assessed using 30 parent-reported items that address the frequency of home learning activities that incorporated math, literacy, and general educational practices in which parents engage with their children. Sample items included “playing board games,” “using number activity books,” “identifying sounds of alphabet letters.” Parents reported on the frequency of the activities using the following response options: 0 = never; 1 = a few times per month; 2 = a few times per week; 3 = every day. An average score of all 30 items was used in analyses (α = 0.89).

### 2.4. Analytic Strategy

Data analyses were completed using Stata 16.0 [49]. The classroom intraclass correlation (ICC) was examined to determine between-classroom variance in order to determine whether multilevel modeling would be appropriate. The ICC was 0.002, and thus our models did not require multilevel modeling. However, to be conservative in our statistical approach, in our regression analyses, we adjusted standard errors to account for clustering at the classroom level. In our analysis, we examined the association between the two indicators of the community food environment (parent reports of the food environment and limited food access) and EF among preschool children, while controlling for the home learning environment, child sex, age, race/ethnicity, and parent education. There were very little missing outcome data (< 5% for HTKS); however, full information maximum likelihood (FIML) was employed to handle missing data.

## 3. Results

### Main Results

Means and standard deviations for all study variables can be found in Table 1. On average, participants scored 8.79 points on the HTKS (*SD* = 13.53). A summary of correlations can be found in Table 2. The HTKS was significantly correlated with parent education (*r* = 0.28, *p* = 0.007). Race was significantly correlated with both limited food access (*r* = −0.23, *p* = 0.040) and reports of the community food environment (*r* = 0.30, *p* = 0.008).

It was hypothesized that higher ratings on both indicators of the community food environment would predict stronger EF skills. Partially as expected, parent reports of the food environment were significantly related to children’s EF (*β* = 0.22, *p* = 0.016) above and beyond limited food access, after controlling for the home learning environment, child sex, child age, race/ethnicity, and parent education. Specifically, a one-unit increase in the community food environment was associated with over three additional points scored on the HTKS. Children normatively gain approximately 1.33 points each month on the HTKS [45]. Thus, what this score indicates is that children experience a fairly substantial increase in EF development (approximately a 3-month gain in EF) with a one-unit increase in parent reports of the food environment. However, contrary to the hypotheses, limited food access was not significantly related to children’s EF skills (*β* = −0.08, *p* = 0.484). Among the control variables, parent education was significantly and positively associated with EF (*β* = 0.23, *p* = 0.023), as well as child sex (*β* = 0.21, *p* = 0.030). See Table 3 for all regression estimates.

## 4. Discussion

The primary goal of this study was to assess whether two indicators of the community food environment (subjective via parent reports of the food environment and objective via limited food access) were related to EF in preschool-aged children. EF skills in preschool are robust indicators of academic [26,28,50], social–emotional [51], and healthy outcomes [25], and EF deficits during early childhood are related to hyperactivity and attention deficits [52]. Thus, identifying early predictors of EF is critical. Results from our study indicated that, after controlling for the home learning environment, child age, race, sex, and parent education, parent reports of the food environment were significantly positively related to stronger EF skills, whereas limited food access was not related to EF. This suggests that the children of parents who believe they have higher quality community food environments have better EF, regardless of whether families are located in census tracts that are flagged as having limited food access.

### 4.1. Community Food Environment and Executive Function

As expected, there was an association between the subjective measure of the community food environment (i.e., parent reports of access to, availability, and affordability of foods) and EF in early childhood. This association may be due to the measure encompassing several environmental barriers to healthful foods that put children at greater nutritional risk, thus affecting developmental outcomes like EF. This link between the community food environment and EF development is novel at the distal ecological level, and variables that only examine limited food access at the tract level, rather than the individual level, may not be able to capture these associations. Food insecurity or insufficiency may be a potential mediating factor between the community food environment and EF. Food insufficiency is defined as inadequate food intake due to a lack of environmental resources, and similarly, food insecurity refers to the limited or uncertain availability of or inability to acquire nutritionally adequate, safe, and acceptable foods due to limited resources which may be impacted by environmental constraints (e.g., the community food environment) [53,54]. Food insufficient/insecure families may live in poor food environments where individuals are 22–35% less likely to have a diet conforming to the DGA than those in food environments with a better availability of supermarkets and healthy foods [13]. Additionally, poverty and environmental impacts of poverty (e.g., akin to social class and environmental impacts), as proposed in the sociocultural layer in the larger ecological model [7], may affect nutrition, and in turn, cognitive development [55]. Food insufficiency/insecurity has a limited but growing literature, showing a link with academic achievement in older U.S. children (e.g., poorer math scores, poorer reading scores, grade repetition [53,54,56]), and EF is closely related to these outcomes [26,50]. Further, previous literature that has directly assessed the concurrent and long-term impacts of food insecurity on EF in preschool-aged (3–5) and early elementary-aged children (6–7) has found that global and domain-specific EFs are significantly negatively impacted when children are exposed to any degree of food insecurity [57,58]. Specifically, one of these studies found that any exposure to any level of food insecurity (either marginally insecure or completely food insecure) in either kindergarten or first grade resulted in worse working memory and cognitive flexibility, two components of EF [57].

Alternatively, there was not an association between an objective measure of the food environment (i.e., limited food access; if at least 500 people within the tract, or 33% of the population were, living more than ½ mile (urban areas) or 10 miles (rural areas) from the nearest supermarket, supercenter, or large grocery store) and EF scores. The finding that parent reports were related to EF and an objective measure of food access was not surprising. This may be because our objective measure of limited food access assumes that (1) full-service grocery stores are a proxy for the presence of affordable and nutritionally sufficient food [37], (2) households buy from the closest supermarket [37], (3) alternative store types may not have a similar selection or may not offer as many fruits or vegetables as supermarkets [59], and (4) families are not getting food from alternative food sources like home and community gardens or farmers’ markets. Moreover, there is evidence of variability within census tracts related to whether individuals with low incomes shop at a neighborhood store or even the nearest chain store [60]. One study found that while SNAP recipients live 1.8 miles on average from full-service grocery stores, most individuals travel 4.9 miles from home to shop for food [61]. Thus, whether a census tract is flagged as limited food access based on distance from a store may not accurately represent individual behaviors regarding where families are going to buy food or access to foods within a store. Furthermore, this measure may not capture small groceries or small general stores.

Additionally, it may be that parent reports of the food environment are more strongly related to cognitive outcomes, like EF. Indeed, parents’ perceptions can be quite powerful influencers on children’s development (e.g., reports of praise and school readiness on academic outcomes, perceptions of being overweight on future weight gain) [62,63]. Furthermore, parent reports of the food environment may tap into an emotional or social connectedness of the community they are in, which may then have an effect on EF outcomes. In one study, social cohesion (sense of belonging and unity among members of a community [64]) and reports of food availability (i.e., a large selection of fresh fruits and vegetables is available in my neighborhood grocery/food stores; a large selection of low-fat products is available in my neighborhood grocery/food stores; the fresh fruits and vegetables in my neighborhood are of high quality [39]) were significantly and positively associated [61].

Furthermore, parents may more accurately predict the community food environment because they may be more aware of their experiences and their access to healthful foods. Census data may not be the strongest indicator of the community food environment because they do not take into account factors that contribute to access, like affordability and transportation. In support of this notion, one scholar has proposed that self-reported levels of constructs, like the community food environment (e.g., food insecurity, hunger, access) may be more appropriate when assessing its relations with outcomes because it truly captures the experiences of the phenomena, as opposed to an indirect indicator, like census data [53].

### 4.2. Limitations and Future Directions

Although this study is the first to document a significant association between parent reports of the community food environment and preschool children’s EF skills, limitations must be noted. The study was limited to just one direct assessment of children’s EF. Future studies would benefit from the use of multiple measures of early EF, as well as the inclusion of more questions about the social, emotional, and other ecological barriers of the community food environment for exploring the extent to which the community food environment may be differentially related to various components of EF. Additionally, there were missing data for the outcome measure (HTKS), parent reports of the food environment, and the limited food access measure. The missingness of the limited food access measure can be attributed to two things: (1) parents did not provide an address or (2) the address provided could not be matched with a census tract using the GIS software. Furthermore, the missingness of the parent reports of the community food environment can be attributed to a lack of response to the question. Although we cannot force research participants to provide responses to survey questions, future research would benefit from full data on the community food environment. Another limitation was that a definition for healthy food was not provided on the parent report of the community food environment, which may have had an impact on how parents responded to items in the survey. It will be important for future studies to include a definition in parent-report measures of the community food environment to ensure consistency in how parents are conceptualizing a healthy diet. Finally, albeit small, the sample was fairly diverse in terms of socioeconomic status (i.e., parent education), but was rather homogeneous in terms of race/ethnicity. A replication of the findings in future studies with larger samples is necessary to ensure generalizability. Future studies should also consider the intermediary role that food security, food insufficiency, and food intake may play between the community food environment and cognitive outcomes.

## 5. Conclusions

Results suggest that researchers need to continue efforts to explore the extent to which the community food environment may be linked with EF and other developmental outcomes in early childhood, at both distal and proximal levels. Policy makers may consider the correlation between the parent reports of the food environment and early EF skills when working to improve current legislation around issues related to community health and well-being. Policy measures that not only improve the community food environment broadly, but also consider and take into account parent perceptions of access, affordability, availability and transportation, may have important health and developmental implications, especially in light of the childhood obesity epidemic that exists in the United States [2]. The current findings also have implications for physicians and pediatricians working with families who may be experiencing barriers to a high-quality community food environment. For example, physicians and pediatricians can consider parent perceptions of access, and could provide additional resources for obtaining access to healthful foods that would help to support children’s healthy development. This research could help set the stage for the development of effective community- and family-based interventions that target improving access to healthful foods by providing a rationale to intervene in communities by linking families with transportation, economical food access, food delivery, and other policies to promote food access. This study also lays a foundation for future research examining the potential impact of poor community food environments and children’s cognitive outcomes.

## Figures and Tables

**Table 1:** Descriptive means and standard deviations for full sample (*N* = 102).

Variable	Mean or % (SD)	Minimum	Maximum
Age (in months)	53.57 (5.42)	40	66
Sex ^a^			
Male (*n* = 48)	48.00%	—	—
Female (*n* = 52)	52.00%	—	—
White (*n* = 71) ^b^	69.61%	—	—
Non-white (*n* = 26)	25.49%	—	—
Parent education ^c^	4.59 (1.59)	1	9
Home learning environment ^d^	2.41 (0.64)	0.80	3.83
HTKS	8.79 (13.53)	0	50
Limited food access ^e^			
Yes limited access (*n* = 28)	34.15%		
No limited access (*n* = 54)	65.85%		
Food environment ^f^	2.85 (0.91)	0	4

*Note.* HTKS = Head–Toes–Knees–Shoulders task. Food environment was measured so that affirmative answers to each of the four questions were scored and tallied with scores ranging from 0–4, where 0 = poor food environment. ^a^ Sex was not reported for two children. ^b^ Race/ethnicity was not reported for five children. ^c^ 1 = 8th grade or less, 2 = some high school, 3 = GED, 4 = high school diploma, 5 = some college, 6 = associate’s degree, 7 = bachelor’s degree, 8 = master’s degree, 9 = doctoral/postgraduate degree. ^d^ Home learning environment was missing for 18 children (84 children). ^e^ Limited food access was missing for 20 children (82 children). ^f^ Food environment was missing for 23 children (79 children).

**Table 2:** Correlation matrix for all study variables (N = 102).

Variable	1	2	3	4	5	6	7
1. Age ^a^	—						
2. Male	−0.14	—					
3. White	0.08	0.19 ^t^	—				
4. Parent education	0.21 *	0.05	0.06	—			
5. Home learning environment	0.06	−0.09	−0.10	0.17	—		
6. HTKS	0.17 ^t^	−0.18 ^t^	0.06	0.28 **	0.12	—	
7. Limited food access	0.01	−0.15	−0.23 *	0.01	0.08	−0.04	—
8. Food environment	0.22 ^t^	−0.00	−0.30 **	0.22 ^t^	0.15	0.21	0.13

Note. ^a^ Child age measured in months. HTKS = Head–Toes–Knees–Shoulders task. ^t^ *p* < 0.10, * *p* < 0.05, ** *p* < 0.01, *** *p* < 0.001.

**Table 3:** Regression estimates predicting executive functioning.

Variable	β (*SE*)
Age ^a^	0.02 (0.10)
Male	−0.21 (0.10) *
White	0.12 (0.10)
Parent education	0.23 (0.10) *
Home learning environment	0.03 (0.08)
Limited food access	−0.08 (0.11)
Food environment	0.22 (0.09) *

*Note*. ^a^ Child age measured in months. * *p* < 0.05, ** *p* < 0.01, *** *p* < 0.001.
